# Investigation of the Damping Capabilities of Different Resin-Based CAD/CAM Restorative Materials

**DOI:** 10.3390/polym14030493

**Published:** 2022-01-26

**Authors:** Thomas Niem, Stefan Gonschorek, Bernd Wöstmann

**Affiliations:** Department of Prosthodontics, Justus-Liebig University, Schlangenzahl 14, 35392 Giessen, Germany; stefan.gonschorek@dentist.med.uni-giessen.de (S.G.); bernd.woestmann@dentist.med.uni-giessen.de (B.W.)

**Keywords:** dynamic mechanical analysis, viscoelastic deformation, plastic deformation, elastic deformation, material damping, energy dissipation, loss tangent, Leeb hardness, CAD/CAM materials

## Abstract

The aim of the present study was to evaluate and quantify the damping properties of common resin-based computer-aided design and computer-aided manufacturing (CAD/CAM) restorative materials (CRMs) and assess their energy dissipation abilities. Leeb hardness (*HLD*), together with its deduced energy dissipation data (*HLD_dis_*), and loss tangent values recorded via dynamic mechanical analysis (DMA) were determined for six polymer, four composite, and one ceramic CRM as well as one metal. Data were statistically analyzed. Among resin-based CRMs, the significantly highest *HLD_dis_* data were detected for the fiber-reinforced composite FD (*p* < 0.001) directly followed by the filler-reinforced Ambarino High Class (*p* < 0.001). The significantly lowest *HLD_dis_* values were observed for the polymer-based CRM Telio CAD (*p* < 0.001). For loss tangent, both PEEK materials showed the significantly lowest data and the polymer-based M-PM the highest results with all composite CRMs in between. *HLD_dis_* data, which simultaneously record the energy dissipation mechanism of plastic material deformation, more precisely characterize the damping behavior of resin-based CRMs compared to loss tangent results that merely describe viscoelastic material behavior. Depending on material composition, resin-based CRMs reveal extremely different ratios of viscoelastic damping but frequently show enhanced *HLD_dis_* values because of plastic material deformation. Future developments in CAD/CAM restorative technology should focus on developing improved viscoelastic damping effects.

## 1. Introduction

Natural bio-composites achieve impressively strong and tough material structures [1] that can repeatedly withstand impact load via damping effects [2]. Producing biomimetic composites with such properties requires the invention of artificial structures that allow viscoelastic material deformation [3] and self-healing effects [4] as much, as early, and as effectively as possible. That is because this type of energy dissipation appears to best preserve structures from destructive effects [5], as it may be repeatedly activated during a material’s lifetime. Although plastic material deformation is also considered to represent an effective element of material damping [6], it has several drawbacks because on the macroscopic scale this effect is not reversible, ruins material dimensions, and cannot be repeatedly activated without any impairments. Conversely, on the microscopic scale and molecular level, plastic material deformation is highly desirable and largely contributes to self-healing and self-repair effects [4]. Therefore, biomimetic restorative materials [7] must be carefully selected to achieve stable and long-lasting results in hard tissue replacement of medical applications where surgical implants are used.

The need for more insight into energy dissipation characteristics to preserve artificial restorations from unwanted destruction effects was reported by Skalak many years ago in the dental profession [8]. He recommended damping on occlusal surfaces for Brånemark implant systems to try to compensate for the energy dissipation capability of the periodontal ligament destroyed due to tooth extraction [9]. Later, an effort was made to technically realize this concept of damping by introducing dampers in implant-supported structures [10]. Unfortunately, the first promising approaches were not consistently and adequately followed up [11]. Therefore, no efficacious restorative materials and methods are available yet that fully allow the periodontal ligament’s natural damping effect to be sufficiently duplicated.

**Figure 1:**
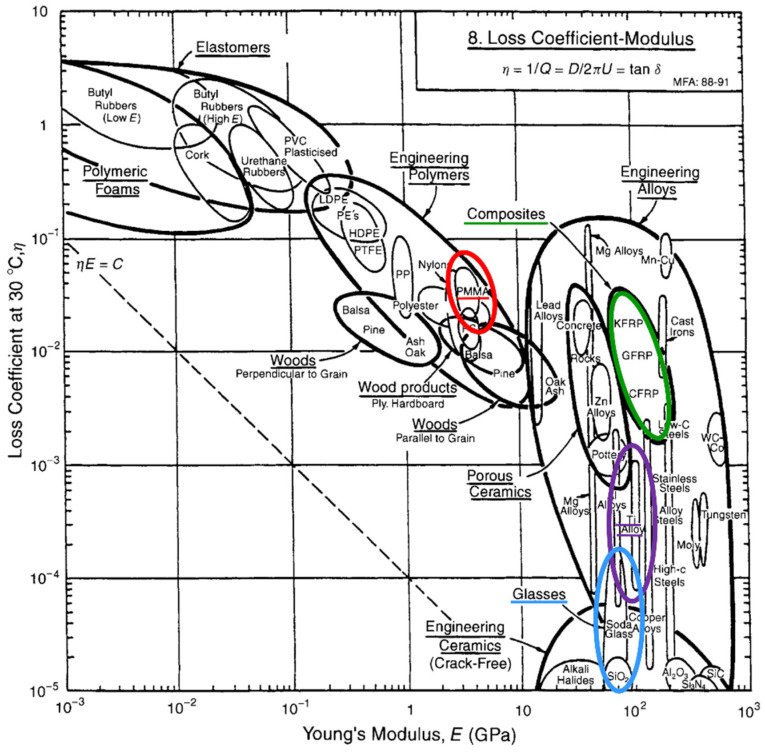
Relationship between Young’s modulus and loss tangent (loss coefficient). Original Ashby diagram [15] (This diagram was published in *Materials Selection in Mechanical Design, Michael F. Ashby, page 432.* Copyright 1999 by Elsevier) Colored cycles represent different material types (red, polymer-based; green, composite; violet, metal and alloys; blue, glass and ceramic).

Today in many clinical situations, accidentally unadjusted artificial restorative materials are used in unfavorable material combinations with a high risk of fracture scenarios. Practitioners have to deal with this phenomenon in their daily work when they observe unexpected material chipping [12], fracture [13], and other destructive processes in places that were technically considered safe and inviolable [14]. A common explanation for this observation is that insufficient attention was paid to the elastic energy transfer to surrounding structures, which is the most ineffective and a physiologically meaningless way of coping with impact load and preserving material structures. This is because the energy is merely passed through different structures and is not effectively attenuated and harmlessly quenched, which is highly desirable from a clinical point of view. Such adverse effects are frequently observed in conjunction with implant-assisted prostheses, and avoiding such effects requires careful restoration planning to fully supplant the natural damping structures as well as possible. Regrettably, during the design of such ultimate restorations for clinical use with the objective of a long-term service time and a warranted survival, different material types are frequently combined to build very strong and resistant constructions. Efforts to solve this challenge often involve the use of restorative materials with a high Young’s modulus such as metals, zirconia, and other ceramics, as this property is usually associated with strength, resistance, and durability. However, it was previously reported that materials with a high Young’s modulus often concurrently demonstrate very low viscoelastic damping properties (loss tangent data), as illustrated by Ashby diagrams (Figure 1) [15]. Consequently, materials with a high Young’s modulus reveal a high preference for elastic energy allocation without any considerable damping effects and so elastically transfer the energy to the immediate vicinity. This has negative implications for vicinal structures, which then have to cope with this unintentional energy impact by means of spontaneous damping (as much as intrinsic material structures allow) or also elastic transfer to the immediate vicinity, or ultimately, in unwanted and senseless self-destruction.

Unfortunately, inelastic material properties such as energy dissipation and damping characteristics are rarely considered during restoration planning and, therefore, disadvantageous material combinations are often selected and inadvertently merged without any knowledge about the load energy attenuation and damping effects of the resulting assembly. One reason for this course of action may be the lack of available information concerning the inelastic material behavior and, accordingly, the damping properties of most single restorative materials. Furthermore, to date, it still appears very difficult to simultaneously determine the elastic and inelastic properties and particularly the relation to one another for one separate material and to reliably compare these results within and between different material classes. Such data and information could be very helpful for practitioners searching for suitable restorative materials and likewise for researchers developing new materials with improved properties. Unfortunately, this kind of information is still missing in common technical specifications. This study therefore aims to provide an initial approach to easily determine the ratio of elastic and inelastic material properties of resin-based computer-aided design and computer-aided manufacturing (CAD/CAM) restorative materials (CRMs), assess their damping capabilities, and estimate the influence on long-term restoration stability. Our previously published method to quantify the damping capability of CRMs via the determination of *HLD* and *HLD_dis_* data appeared to be appropriate for use in the present investigation [16].

Since a meaningful comparative evaluation of the inelastic material properties of resin-based CRMs is not available yet, this in vitro study aimed to investigate the *HLD_dis_* data and loss tangent values (dynamic mechanical analysis (DMA)) determined for different resin-based CRMs. The null hypothesis tested was that *HLD_dis_* values and loss tangent data are independent of the CRM and material type tested.

## 2. Materials and Methods

### 2.1. Materials

For this study, ten commonly used resin-based CRMs were selected and also one metal and one ceramic CRM as comparative material reference (Table 1). VCT, CPw, and DMG-P were classified as CAD/CAM polymers following a previous suggestion [17], knowing that these CRM contain low ratios of filler particles (Table 1).

### 2.2. Specimen Preparation

For DMA investigations, bar-shaped specimens (16.00 mm × 4.00 mm × 1.00 mm ± 0.05 mm, *n* = 10) were prepared for each CRM, while for *HLD_dis_* determinations, square-shaped specimens (12.0 mm × 12.0 mm × 3.5 mm ± 0.1 mm, *n* = 2) were produced. All materials were used under ambient laboratory conditions (23 ± 1 °C; 50 ± 5% relative humidity) and according to the manufacturer’s user instructions. Printed materials were provided by the manufacturer in block shape geometry and processed in the same way as all resin-based CRMs. A water-cooled precision saw (IsoMet™1000, Buehler, Lake Bluff, IL, USA) in combination with a diamond-coated saw blade (127 mm diameter, No. 11-4255, Buehler, Lake Bluff, IL, USA) was used for resin-based CRMs as well as ceramics to cut off milling block mounts and slice blocks or discs to the desired dimensions before grinding. Metal specimens with the desired dimensions were milled in accordance with the respective manufacturer’s instructions (cara Mill 3.5 Pro milling machine, Kulzer GmbH, Hanau, Germany). All final specimen surfaces were polished by grinding on wet SiC paper using P1200, P2500 (LECO Corporation, St. Joseph, MI, USA), and P4000 (Struers A/S, Ballerup, Denmark) in succession, and dimensions were controlled with a digital outside micrometer (Type 293-521-30, Mitutoyo, Kawasaki, Japan; accuracy ± 1 µm).

### 2.3. Water Storage

Specimens from each CRM and for every test procedure (*HLD_dis_* and loss tangent) were stored in distilled water (Aqua B. Braun, B. Braun Melsungen AG, Melsungen, Germany) using separate glass vessels that were placed in an incubator (Ehret, Emmendingen, Germany) at 37 °C for 24 h. For DMA measurements, specimens were removed one by one and placed directly on the lower support bars in the DMA specimen holder and fixed via the upper support clamp. For *HLD_dis_* determinations, the specimens were removed and placed for one minute at ambient laboratory conditions on a tissue paper for drying. Measurements were carried out immediately without any further delay and treatment.

### 2.4. Leeb Hardness Determination

**Figure 2:**
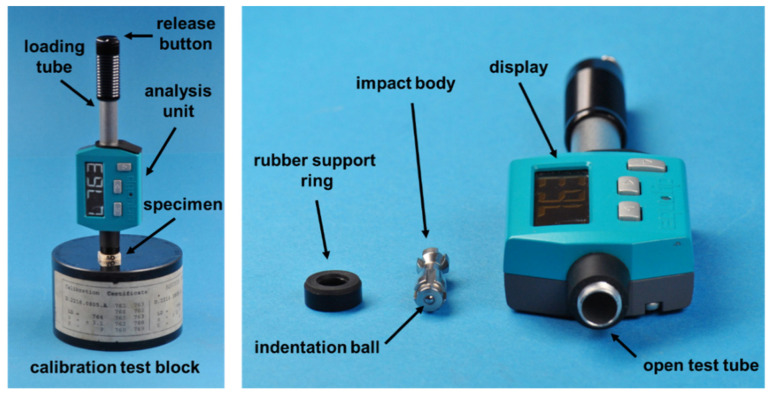
*HLD* test device (Equotip Piccolo 2, Proceq SA, Schwerzenbach, Switzerland). **Left** side: Device placed together with specimen on the calibration test block; **right** side: disassembled *HLD* test device.

The *HLD* test device (Figure 2) used was newly calibrated by the manufacturer, who provided a calibration certificate. All indentations were conducted under ambient laboratory conditions (23 ± 1 °C; 50 ± 5% relative humidity) with the device-specific spherical indenter made of tungsten carbide (1500 HV). Before measurements were performed on specimen surfaces, the device accuracy was carefully controlled as recommended by the manufacturer via ten test indentations directly performed on the calibration test block shown in Figure 2 [18]. The calculated mean revealed a maximum deviation of six HLD-units (0.80%) when compared to the certified value specified on the calibration test block.

*HLD* of CRM was determined by centrically positioning each specimen (see Section 2.3) on the calibration test block, which was placed on a 10 mm thick granite plate. The indentation process was carried out as described in the related ISO standard [18] and the respective user instructions [19]. Five indentations were performed on the same specimen surface with the arrangement very closely resembling a five depicted on dice to ensure the required distances specified in the ISO standard [18]. Two specimens were examined for each CRM, except for VB, where one specimen was used for each measurement due to a previously observed high fracture risk.

The dimensionless *HLD* values were directly read off from the device display and were internally calculated via the device software like previously explained [20] and defined [18]:

$$HLD=\frac{\nu_{R}}{\nu_{A}}\times 1000$$

with *ν_R_* (rebound velocity) in m/s and *ν_A_* (impact velocity) of 2.1 m/s.

Consequently, the amount of energy that was logically lost (converted and dissipated) on the material surface and not possible to recover was simply deduced from *HLD* by means of subtracting *HLD* values from 1000. As previously explained [21], the respective results were defined as *HLD_dis_* in this publication to describe energy dissipation effects:

$$HLD_{dis}=1000-HLD$$

Furthermore, to be able to roughly characterize the viscoelastic damping part of *HLD_dis_* values, its results were related to loss tangent data and consequently split into *HLD_dis loss tangent_* and *HLD_dis residue_*:

$$HLD_{dis}=HLD_{dis\;loss\;tangent}+HLD_{dis\;residue}$$

As no permanent plastic material deformation and signs of destruction could be observed for M-PM directly after HLD determination (*HLD_dis residue M−PM_* = 0), *HLD_dis loss tangent M−PM_* was equal *HLD_dis M−PM_* (*HLD_dis M−PM_* = 151.0), assuming that *HLD_dis M−PM_* consists of 100% viscoelastic deformation. This value and the respective *loss tangent_M−PM_* = 0.1414 were defined as a reference and used to calculate and approximate all *HLD_dis loss tangent_* data in relation to this data set:

$$HLD_{dis\;loss\;tangent\;CRM}={\frac{HLD_{dis\;loss\;tangent\;M-PM}\times loss\;tangent_{CRM}}{loss\;tangent_{M-PM}}}_{\;}$$

### 2.5. Dynamic Mechanical Analysis (DMA)

**Figure 3:**
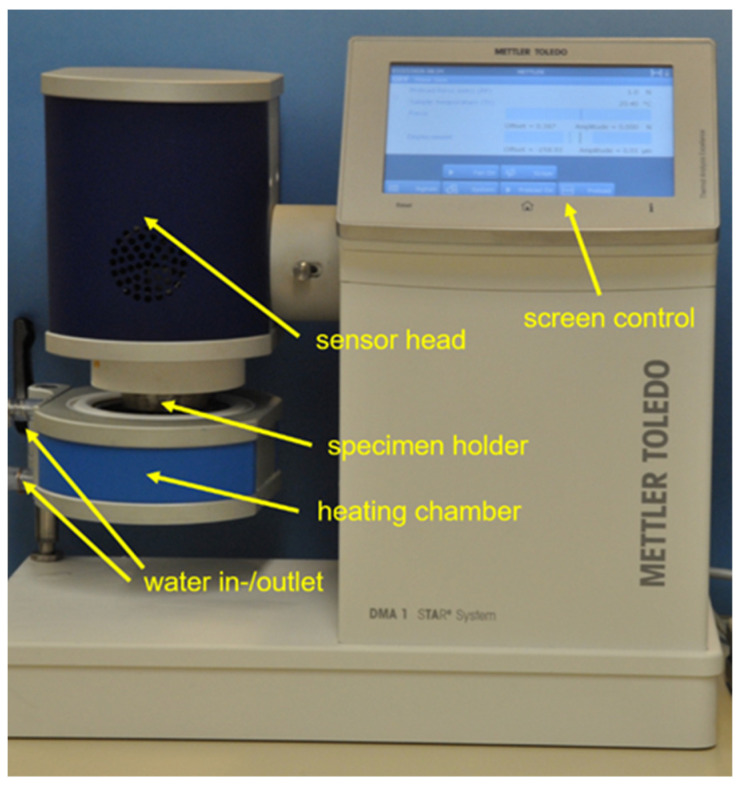
Dynamic mechanical analyzer (DMA 1) with heating chamber and specimen holder.

For ten specimens, loss tangent values were determined using a dynamic mechanical analyzer (DMA 1, Mettler-Toledo GmbH, Giessen, Germany) in combination with an appropriate double-walled heating chamber filled with distilled water (Aqua B. Braun, B. Braun Melsungen AG, Melsungen, Germany) (Figure 3). The heating chamber was warmed up using an external water bath (Polystat Control cc1, Huber GmbH, Offenburg, Germany) to ensure a consistent water temperature of 37.0 ± 0.5 °C inside the chamber. The water temperature was carefully monitored via the system-integrated sensor. All DMA measurements were performed in the ‘three-point bending mode’ using customized lower support bars with a support distance in the device of 14.85 mm to also allow a reliable and comparable investigation of specimens received from limited block dimensions. Specimens were centrically clamped between the three support bars with a constant pre-deformation of 158 ± 2 µm to ensure a permanent specimen contact during the cyclic load application of the test procedure. After mounting the specimen, the DMA instrument’s specimen holder was immediately immersed into the water of the heating chamber to avoid unintended drying out and cooling down of the specimen. The sealed system was allowed to equilibrate for at least 10 min to reach a temperature of 37.0 ± 0.5 °C before the measurement was started. A sinusoidal dynamic test load of 1 N was applied to each specimen with 1.5 Hz during an investigation period of 10 min. This frequency was used to resemble previously determined average chewing rates in the natural environment as closely as possible (1.58 Hz [22], 1.33 Hz [23], 1.48 ± 0.18 Hz [24], and 1.57 Hz [25]). Sixty loss tangent values (tan δ) were generated per specimen and analyzed via the belonging system software and subsequently averaged (STARe System, Mettler-Toledo GmbH, Giessen, Germany).

A detailed introduction into DMA technology and data acquisition is presented by Menard [26]. In general, loss tangent is defined as:

$$\tan\delta =\frac{E^{\prime \prime }}{E^{\prime }}$$

where tan *δ* (dimensionless) is the loss tangent (damping factor describing the ratio of loss modulus and storage modulus with *δ* representing the phase lag between the applied stress and the resulting strain in a viscoelastic material), *E*′′ is the loss modulus (material ability to lose/dissipate energy, representing the viscous component of a viscoelastic material), and *E*′ is the storage modulus (material ability to store / return energy without phase difference between stress and strain, representing the elastic component of a viscoelastic material). Both moduli were determined by the DMA instrument as the answer of the tested material to an oscillating loading condition. They were recorded simultaneously and tabulated in the system software.

### 2.6. Surface Topography, Indentation Depth, and Indentation Volume

Surface topographies and indentation depths and volumes were analyzed with an optical profilometer (MicroProf 200, Fries Research & Technology GmbH, Bergisch Gladbach, Germany). A 600 µm sensor with a precision of 200 nm and a vertical resolution of 20 nm was used, and the investigated area was 2.50 mm × 2.50 mm in size (sensor frequency 1000 Hz). Surface profiles were optically analyzed using the accompanying Mark III Software (release 3.11.5.2, Fries Research & Technology GmbH, Bergisch Gladbach, Germany), and raw data were treated with a software-specific smoothing function before measurement. Three independent measurements were, respectively, performed to calculate the means of the indentation volume (10^4^ µm^3^) and indentation depth (µm) and not earlier than after 24 h storage time at ambient laboratory conditions to account for viscoelastic relaxation processes.

### 2.7. Statistical Analysis

Mean values and standard deviations of loss tangent and *HLD_dis_* data were calculated. Normality of data distribution was analyzed using the Shapiro–Wilk test and the homogeneity of variances was checked using the Levene test. As the test results revealed, although there was no group that deviated significantly from the normal distribution, no homogeneity of variance was observed. Multiple comparisons of different CRMs were analyzed using one-way analysis of variance (ANOVA), and post hoc comparisons were carried out using a Games–Howell test. All statistical analyses were carried out using IBM SPSS Statistics for Windows (version 23.0.0.2, IBM World Trade Corporation, Armonk, NY, USA) at a significance level of 0.05. Bar chart diagrams were prepared with Excel (Office 16, Microsoft Corporation, Redmond, WA, USA).

## 3. Results

**Figure 4:**
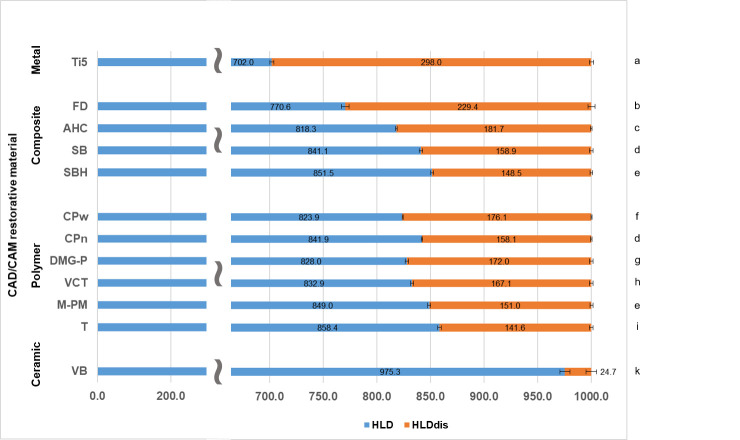
*HLD* and *HLD_dis_* data determined via Leeb hardness determination. For *HLD*, the scaling was interrupted (blue bars) to allow a more precise interpretation and view on significantly different *HLD_dis_* values. Identical lowercase letters on the right side denote no significant difference among CRMs (*p* > 0.05). Parts of this data for Ti5, AHC, SB, SBH, VCT, M-PM, T, and VB were published in Journal of the Mechanical Behavior of Biomedical Materials, Th. Niem, S. Gonschorek, B. Wöstmann, Evaluation of the damping capacity of common CAD/CAM restorative materials, https://doi.org/10.1016/j.jmbbm.2021.104987 accessed on 17 January, 2022, Copyright Elsevier 2022.

Figure 4 shows the results of the Leeb hardness determination, simultaneously displaying the measured HLD and calculated *HLD_dis_* data of the present study. Both selected reference materials define the significantly highest (Ti5: 298.0, *p* < 0.001) and significantly lowest (VB: 24.7, *p* < 0.001) *HLD_dis_* data sets. Among the tested resin-based CRMs, the fiber-reinforced composite FD revealed the significantly highest damping effect (*p* < 0.001), showing a *HLD_dis_* value of 229.4, which was closest to the results of the metal Ti5 (298.0). With the filler-reinforced composite AHC, the third-highest damping result (*HLD_dis_* = 181.7) was obtained in this study, which appeared to be significantly higher than the data sets of all the other resin-based CRMs (*p* < 0.001). The significantly lowest *HLD_dis_* data among the resin-based CRMs and with that the least damping effects were observed for the polymer-based material T (*p* < 0.001).

**Figure 5:**
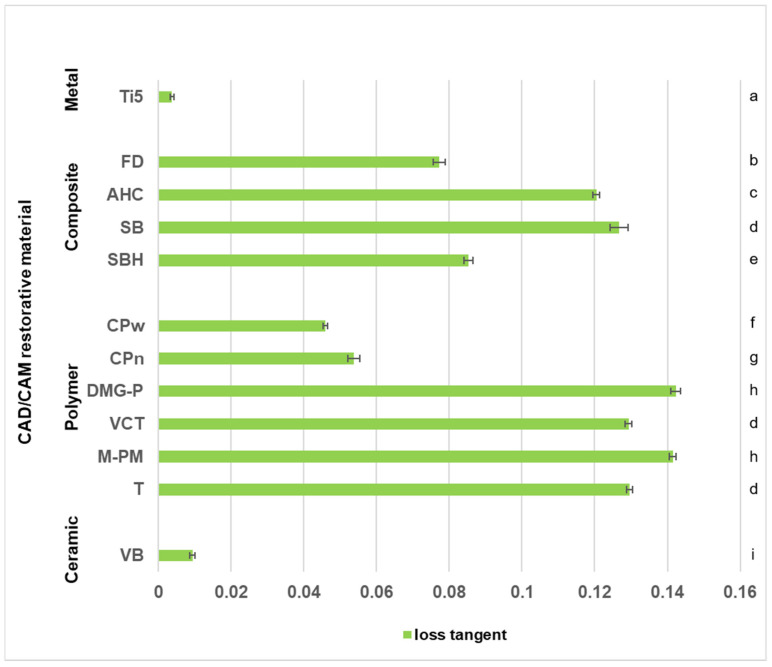
Loss tangent data determined via dynamic mechanical analysis. Identical lowercase letters on the right side denote no significant difference among CRMs (*p* > 0.05). Parts of this data for Ti5, AHC, SB, SBH, VCT, M-PM, T, and VB were published in Journal of the Mechanical Behavior of Biomedical Materials, Th. Niem, S. Gonschorek, B. Wöstmann, Evaluation of the damping capacity of common CAD/CAM restorative materials, https://doi.org/10.1016/j.jmbbm.2021.104987, accessed on 17 January, 2022, Copyright Elsevier 2022.

Means and standard deviations of loss tangent values were calculated for each CRM and displayed in Figure 5. Data sets were arranged according to the different material types. Among polymer CRMs, T, M-PM, VCT, and DMG-P showed significantly higher viscoelastic damping effects than the two PEEK-based polymers (CPn and CPw) with M-PM and DMG-P not being significantly different (*p* = 0.995) but with both simultaneously revealing the highest loss tangent values observed in the present study (*p* < 0.001). Fiber- and filler-reinforced composites show loss tangent values in between both PEEK-based polymers and the other resin-based polymer materials, with SB not being significantly different from VCT (*p* = 0.670) and T (*p* = 0.540). The significantly lowest results for loss tangent were received for the two reference materials, metal (Ti5) (*p* < 0.001), and ceramic (VB) (*p* < 0.001). Consequently, both revealed the lowest viscoelastic damping properties observed in the present investigation.

**Figure 6:**
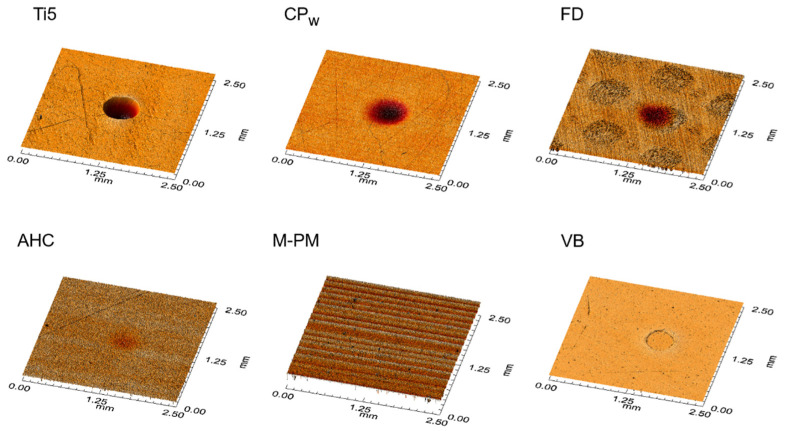
Representative surface topography images of *HLD* indentation areas. Indentations represent different permanent plastic material deformation of CRMs. Note greyish circular structures for FD, which represent glass fiber bundles of the composite material.

Selected and representative surface topographies to characterize the Leeb hardness indentation areas are displayed in Figure 6, while the respective indentation volumes and depths are summarized in Table 2. The deepest indentations were observed for Ti5 followed by the PEEK-based polymers CPn and CPw as well as the fiber-reinforced composite FD. In contrast to this ranking, no indentation or further surface alterations were detected for all the other polymer CRMs (DMG-P, M-PM, T, and VCT) (Table 2).

## 4. Discussion

The hypothesis that the *HLD_dis_* values and loss tangent data are independent of the CRM and material type tested could only partially be rejected.

**Figure 7:**
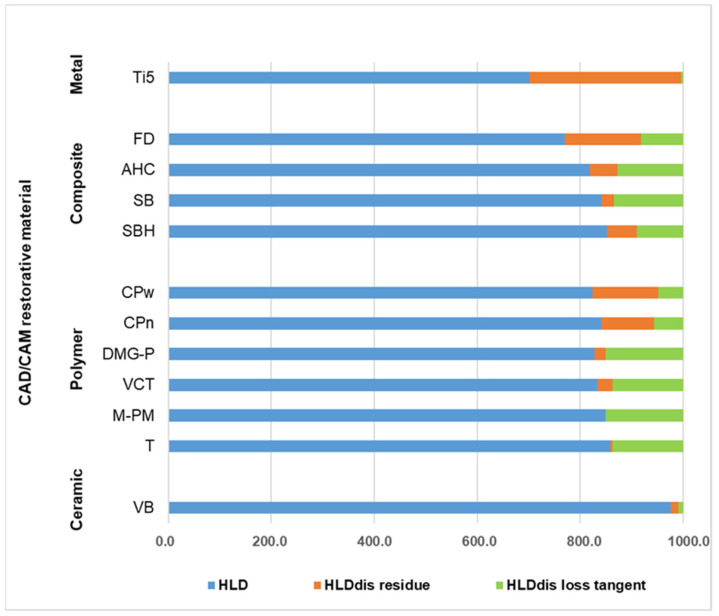
Devised diagram to facilitate interpretation of energy allocation during impact load. Figure 4 was used as a basis and all lengths of green bars were calculated defining the determined loss tangent values of M-PM as 100% *HLD_dis_*.

A reasonable interpretation of the different damping effects and impact energy allocations in resin-based CRMs is possible if the results received from *HLD/HLD_dis_* determination and DMA measurement are considered together. Therefore, Figure 7 was devised to graphically depict the information acquired from these data in one figure and to allow a more profound and differentiated comparison of the determined damping properties. In Figure 7, *HLD* values were displayed in the same way as in Figure 4 but with the main difference that *HLD_dis_* results were further split into *HLD_dis loss tangent_* and *HLD_dis residue_*, whose values were calculated as described in Section 2.4. The result of this data transformation shows green bars representing *HLD_dis loss tangent_* roughly characterizing the viscoelastic damping part of *HLD_dis_*. Orange bars in the same diagram describe the residual *HLD_dis_* values (*HLD_dis residue_*) being attributed to either permanent plastic material deformation or other energy dissipation mechanisms. Similarly, Vaidyanathan et al. separately analyze the elastic, viscoelastic, and viscous (permanent plastic deformation) ratio of deformation compared to the total material deformation determined [27]. Figure 7 may therefore make it easier to understand deviant material damping behavior from a deeper materials science point of view.

One essential mechanism to dissipate impact energy and to induce material damping is the well-known effect of permanent plastic material deformation. Figure 6 displays the topographic images of representative *HLD* indentation areas demonstrating the phenomenon of plastic deformation, with Ti5 as reference material showing the deepest indentation of 13.42 µm (Table 2). This result implies that Ti5 has the highest allocation of impact energy to permanent plastic material deformation in this comparison. However, at the same time, a very low viscoelastic damping effect was observed for this metal, which is demonstrated by its comparatively low loss tangent values in the present study (Figure 5). These are perfectly in line with recently published results determined at room temperature for a Ti-6Al-4V alloy (>0.0026) [28] and for grade 4 Ti (<0.010) [29]. The different results of energy allocation for Ti5 during impact load are schematically shown in Figure 7. This metal reveals the overall highest rate of impact energy dissipation in this material comparison (highest *HLD_dis_* = 298.0 = 1000.0 − 702.0; Figure 4) and hence the highest damping effect, with almost all of the dampened energy being dissipated and transferred on the surface to permanent plastic material deformation. Nevertheless, 70.2% (702/1000 × 100; Figure 4) of the total impact energy impinging on this metal is still converted elastically without any efficacious damping effect. The relatively low damping effects of pure metals have already been discussed [30], and metal–matrix composites have been described as revealing improved and promising properties [30]. Apart from that, the highest rate of impact energy dissipation among all investigated resin-based CRMs of this study was observed for the fiber-reinforced composite material FD (*HLD_dis_* = 229.4 = 1000.0 − 770.6; Figure 4). Although the damping properties of this material are closest to those of the metal, different mechanisms of material damping appear to be present. As demonstrated via DMA results, and in comparison to Ti5, a considerable amount of impact energy is consumed via riskless viscoelastic deformation (Figure 5). However, some energy is also dissipated via permanent plastic material deformation, as demonstrated by a deep indentation area (2.37 µm) observed after *HLD_dis_* determination (Table 2). With this behavior, the fiber-reinforced resin-based FD most closely resembles Ti5, which is clinically accepted for use as a restorative material in prosthetic dentistry [31] and total hip replacement [32].

Conversely, M-PM, as a member of pure polymer-based CRMs, showing only the ninth-highest rate of energy dissipation (*HLD_dis_* = 151.0 = 1000.0 − 849.0; Figure 4), reveals a completely different allocation mechanism that obviously dissipates energy acting on surface structures entirely via viscoelastic material behavior (high loss tangent, Figure 5), as no permanent plastic material deformation could be observed after Leeb indentation (Figure 6). This comparatively high damping capability (highest loss tangent value, Figure 5) with almost no permanent plastic deformation may help to explain the superior performance of polymethylmethacrylate-based fixed dental prostheses as long-term temporaries in vivo [33] and in vitro [34]. This is because destructive impact energy is mainly accommodated by compliant behavior through riskless viscoelastic deformation resulting in a beneficial damping effect. Hence, this material characteristic represents a perfect form of structure preservation without the risk of permanent deformation or unwanted fracture scenarios and is likewise adopted by the other polymer CRMs T, DMG-P, and VCT. In contrast, a markedly different behavior was observed for the PEEK-based polymers CPw and CPn, which appear to have similar *HLD_dis_* values but different energy dissipation mechanisms than the other polymer CRMs. While viscoelastic deformation seems to be the main mechanism of these polymers, this cannot be found to such an extent for the PEEK-based CRMs CPw and CPn (Figure 5), where, instead, a distinct permanent plastic deformation is present, which is obvious when investigating the surface indentations after *HLD* determination (Figure 6). Rather deep indentations of 3–5 µm were observed in comparison (Table 2) demonstrating that approximately twice the amount of energy is dissipated via plastic deformation when compared to viscoelastic deformation (Figure 7). Unfortunately, there is still little information available about whether this considerable plastic deformation of PEEK-based polymers under load is desirable from a clinical point of view when these are used as a restorative material in prosthodontic dentistry [35] and other biomedical applications [36]. Further clinical studies in this field of investigation, especially in direct comparison to PMM-based polymers that show a high proportion of viscoelastic deformation, would be highly appreciated.

In contrast to the energy dissipation mechanisms of the aforementioned CRMs containing no or only low ratios of reinforcing fillers (Table 1), for ceramic materials like VB, also used as reference material in this investigation, none of these energy dissipation effects appear to be available. Therefore, these and similar CRM material structures based on ceramics appear to be comparatively vulnerable to force overload, which often results in sudden and unexpected material destruction [37]. Hence, the only way for ceramic CRMs to cope with potential excessive stresses is to elastically (VB: 97.5% = 975/1000 × 100; Figure 4) transfer this impact energy to the surrounding structures. These structures then have to be able to cope with this challenge or self-destruct, as previously demonstrated by VB [21] and clinically observed on zirconia implants [38] restored with single crowns made of feldspathic ceramics [39].

In between these extremes of energy dissipation effects represented by metals (permanent plastic deformation), polymers (viscoelastic deformation), and ceramics (elastic transfer or self-destruction), there is the well-placed material type of filler-containing composites. AHC, SB, and SBH are exemplary composites with high filler content, as shown in Table 1. All three simultaneously demonstrate an optimal balance of the previously discussed mechanisms of viscoelastic and permanent plastic material deformation (Figure 7) to attain a maximum effect of energy dissipation. The highest damping effect was achieved by AHC (*HLD_dis_* = 181.7 = 1000.0 − 818.3, Figure 4) even though a high filler content of 68.3% was determined. In comparison to polymer-based CRMs, which have a markedly lower filler content (Table 1) and nonetheless exhibit similar *HLD_dis_* results, filler-containing composites possess the crucial advantage of a higher abrasion resistance [40] and therefore show a higher prognosis for long-term material survival in clinical applications.

Nevertheless, all three composites together with the fiber-reinforced FD reveal a significantly higher proportion of viscoelastic material deformation if compared to the ceramic VB and even the PEEK-based polymers (Figure 5), which results in a clearly higher total damping effect than VB. Consequently, AHC has the third-highest rate of energy dissipation in this investigation (*HLD_dis_* = 181.7), directly following FD (*HLD_dis_* = 229.4 = 1000.0 − 770.6, Figure 4). From a clinical viewpoint, this well-balanced combination of viscoelastic and permanent plastic material deformation, as demonstrated by further filler-containing (BRILLIANT Crios (Coltène/Whaledent AG, Altstätten, Switzerland), CERASMART (GC Germany GmbH, Bad Homburg, Germany), Lava Ultimate (3M Oral Care, St. Paul, MN, USA), Tetric CAD (Ivoclar Vivadent AG, Schaan, Liechtenstein), Grandio blocs (VOCO GmbH, Cuxhaven, Germany)) and fiber-reinforced (TRINIA (bicon, Boston, MA, USA) CRMs, appears to be highly desirable, since energy dissipation effects realized via viscoelastic deformation may be frequently activated and harmlessly repeated several times during a material’s life of service without any adverse material interference [41]. This effect was recently observed for an in vitro study, which investigated the fatigue performance of adhesively cemented CRMs where only a resin nanoceramic material survived with 100% probability, showing solely occlusal deformation instead of cracks and failures, which were observed for ceramic-based materials in the same test situation [42]. On the contrary, damping via permanent plastic deformation may be activated only once, and just a small ratio realized in clinical practice is attributed, for example, to seal gaps and margins in gold [43] and amalgam restorations [44]. However, if it is implemented to a large extent, it may cause material fit problems and, consequently, induce restoration loosening effects and microleakage [45].

The positive damping effect of composite materials might also have contributed to the superior fracture resistance results of occlusal all-ceramic restorations made from lithium disilicate ceramic luted on prepared enamel/dentin molar surfaces that were previously restored with composite. If these were directly compared to similar restorations prepared on the basis of pure enamel surfaces, significantly higher fracture results were observed for the composite version independent of the restoration thickness [46]. As revealed in the present study, in this special case, the lithium disilicate ceramic would have elastically transferred the entire fracture load to vicinal structures without any remarkable damping effect, where the material structures of composite and dentine have markedly higher damping and energy dissipation capabilities than pure enamel. Consequently, the structures made of composite and dentine appear to show higher fracture forces. A similar effect of the supporting structure was recently disputed for Vita Enamic but was related solely to the elastic modulus and not to the damping characteristics of CRMs [47]. Therefore, further studies investigating the energy dissipation effects of single materials and especially their influence in multilayered assemblies, resembling clinical situations as close as possible, should be devised and performed to discover new damping effects with the aim of preserving structures from undesired fracture scenarios. In these experiments, it should be systematically examined what influence different or similar elastic moduli of the layering materials have on the total damping effect of the system. The question arises whether the cases of modulus match or mismatch would improve the total damping effect provided that the properties of the luting material layer were kept constant. Further studies that investigate this relationship are already under consideration in our labs.

For all CRMs investigated under the conditions of the present study, the majority of the impact energy (>70%; *HLD*/1000 × 100; blue bars Figure 4) acting on the surface structures is still allocated elastically and merely transferred to the surrounding structures without undergoing any relieving damping processes. Therefore, CRM manufacturers might be requested to develop new and more biomimetic materials in which the current proportion of 70% elastic energy allocation during impact load is considerably reduced and where intrinsic material properties are implemented that instead maximize riskless viscoelastic deformation, since this characteristic is not yet sufficiently realized in CRMs and exploited during energy allocation after load impact. Especially fiber-reinforced CRMs such as FD appear to be a very promising material class with high potential for damping property improvement. From a chemical point of view, these materials provide several tuning parameters to realize specific designs, which further optimize energy dissipation processes and therewith enhance damping characteristics. In this way, new artificial restorative materials with improved long-term survival rates may be developed for medical applications.

As standard testing procedures, such as conventional hardness test methods and strength data determinations, do not provide sufficient information and data to reliably assess damping characteristics, the DMA and *HLD_dis_* measurement methods presented in this study constitute very helpful approaches to soundly identify new materials with optimized damping characteristics under simulated clinical conditions. Nevertheless, *HLD_dis_* appears to be more effective for characterizing total material damping because plastic material deformation is captured and also recorded with this method. DMA data determination and its parameters are not able to adequately describe plastic deformation and therewith often fail to reliably evaluate total damping effects.

## 5. Conclusions

If they are investigated separately, loss tangent and *HLD_dis_* values allowed a distinct differentiation of the damping capabilities of different resin-based CAD/CAM restorative materials. However, the principles underlying the measurements for the two methods differ considerably. While loss tangent data only describe and record viscoelastic material properties determined in the linear elastic region of damping effects, *HLD_dis_* data also take into account the damping phenomenon of plastic material deformation, which is considered to be an effective element of damping mechanisms too, especially on the microscale and molecular level. This difference is extremely important when comparing various resin-based material types in terms of their damping ability, as polymer structures are considered to substantially affect viscoelastic material behavior. The results of our present investigation reveal that:

1. Resin-based CRMs show damping capabilities (*HLD_dis_*) being categorized in between metals (Ti5) and ceramics (VB).
2. For resin-based CRMs, most impact energy (>75%) is still converted elastically (*HLD*) and so merely transferred to the surrounding structures without any relieving damping processes.
3. Different polymer structures such as PEEK and the other polymer-based CRMs of the same group show significantly different loss tangent values and so a deviant viscoelastic material behavior. However, at the same time, they also reveal very similar *HLD_dis_* data as a consequence of divergent plastic material deformation.
4. Fiber-reinforced composite materials (FD) show a well-balanced mixture of viscoelastic (loss tangent) and plastic material deformation and so the highest damping capacity (*HLD_dis_*) of all investigated resin-based CRMs. FD is closest to the results of the metal Ti5.
5. Although composite CRMs exhibit markedly higher filler contents (>60%) compared to the polymer-based CRMs, they still reveal a high proportion of viscoelastic material deformation (loss tangent) and so similar damping properties (*HLD_dis_*).

As a general result, it can be stated that composite CRMs reveal a high potential for focused damping improvement, especially if fiber-reinforced materials are considered. One aim in future CRM developments should be to reduce the currently still high amount of energy that is converted elastically to the surrounding structures without any effective and relieving damping effect during impact load. Implementation of enhanced viscoelastic material properties appears to be a key step in this regard.

## Figures and Tables

**Table 1:** Tested materials.

Material Type	Brand	Code	Manufacturer	Lot No. ^$^	Filler Content/% ^#^
Ceramic	VITABLOCS Mark II 2M2C	VB	VITA Zahnfabrik	57000 (b)	99.8
Composite	AMBARINO High Class A2	AHC	Creamed GmbH	160117 (b)	68.3
	Shofu Block HC A2 LT	SB	SHOFU Inc.	0819927 (b)	61.8
	Shofu Block HC hard A2 LT	SBH	SHOFU Inc.	0819912 (b)	68.5
	Fiber Disc ZH	FD	bioloren srl	1302 (d)	64.0
Polymer	Ceramill PEEK Oyster white	CPw	Amann Girrbach AG	M000714 (d)	10.3
	Ceramill PEEK natural	CPn	Amann Girrbach AG	M000794 (d)	0.1
	Experimental print material	DMG-P	DMG GmbH	X35-F0006-C (p)	30.7
	M-PM Disc A2	M-PM	Merz Dental GmbH	10417 (d)	0.1
	Telio CAD A3 LT	T	Ivoclar Vivadent AG	VY0857 (b)	0.1
	Vita CAD-Temp 1M2T	VCT	VITA Zahnfabrik	23170 (d)	14.5
Metal	Starbond Ti5 ^a^	Ti5	S&S Scheftner GmbH	5024071119 (d)	-----

^$^ b, block; d, disc; p, printed. ^#^ Ashing in air (3 h, 800 ± 20 °C, n = 3); ^a^ Grade 5 (ELI), Ti: 89.4 wt%, Al: 6.2 wt%, V: 4.0 wt%, N, C, H, Fe, O < 0.4 wt%.

**Table 2:** Characteristics of Leeb indentations.

Material Type	CRM	Volume/10^4^ µm^3^	Depth/µm
Ceramic	VB	1.11	0.13 ^#^
Composite	AHC	1.06	0.67
	SB	1.71	0.55
	SBH	4.72	1.14
	FD	17.20	2.37
Polymer	CPw	32.74	3.29
	CPn	55.12	4.40
	DMG-P	0.00	0.00
	M-PM	0.00	0.00
	T	0.00	0.00
	VCT	0.00	0.00
Metal	Ti5	120.60	13.42 ^#^

^#^ depth measured from the surrounding flat surface as reference not considering the indentation rim. CRM, CAD/CAM restorative material.

## Data Availability

Data available on request due to privacy.
